# Dural masses: meningiomas and their mimics

**DOI:** 10.1186/s13244-019-0697-7

**Published:** 2019-02-06

**Authors:** Daniel Lyndon, Joseph A. Lansley, Jane Evanson, Anant S. Krishnan

**Affiliations:** Department of Neuroradiology, St Bartholomew’s and the Royal London Hospitals, Whitechapel, London, E1 1BB UK

**Keywords:** Diagnosis, Differential, Meningioma, Meningeal neoplasms, Dura mater, Imaging, Diagnostic

## Abstract

Meningiomas are the most common dural tumour. They are regularly being seen as an incidental finding on brain imaging and treated conservatively. However, there are many other dural masses which mimic their appearances, including primary neoplastic processes, metastases, granulomatous diseases and infection. While some of these are rare, others such as metastases and tuberculosis arise relatively frequently in practice. Although not pathognomonic, key features which increase the probability of a lesion being a meningioma include intralesional calcifications, skull hyperostosis, local dural enhancement and increased perfusion. It is important to have an awareness of these entities as well as their main imaging findings, as they have a wide range of prognoses and differing management strategies. This review outlines several of the most important mimics along with their imaging findings on both standard and advanced techniques with key features which may be used to help differentiate them from meningiomas.

## Keypoints

- A range of pathologies cause meningeal thickening or masses, therefore mimicking meningiomas.
- Meningiomas commonly calcify and cause bony hyperostosis.
- Many mimics lack calcification and result in destruction or erosion of the bone.
- A dural tail is not pathognomonic of meningioma, occurring in many mimics.
- Meningiomas have higher perfusion except for solitary fibrous tumours and hypervascular metastases.

## Introduction

Meningiomas are the most common mass lesion of the dura, accounting for 38% of intracranial tumours in women and 20% in men [1]. However, there are a number of other dural lesions mimicking their imaging appearances with considerably different prognoses and management strategies. Data regarding the prevalence of these lesions is limited, with most examples in the literature existing in case reports and small case series. The largest study of around 1000 cases found 2% of resected dural masses initially diagnosed as meningiomas are found to be other pathologies [2]. These may include metastatic disease, solitary fibrous tumours (SFT) and melanoma, as well as non-neoplastic processes such as tuberculosis (Table 1).

**Table 1:** Meningioma mimics in the literature

Meningioma mimics	
Neoplastic:	
Metastasis	
Solitary fibrous tumour	
Melanocytic tumours	
Glioblastoma	
EBV-associated smooth muscle tumours	
Granulomatous:	
Tuberculosis	
Granulomatosis with polyangiitis	
Sarcoidosis	
Lymphoproliferative	
Lymphoma	
Rosai-Dorfman disease	
Erdheim-Chester disease	
Autoimmune	
IgG4-related disease	

Despite being infrequent, an awareness of these mimics is important. For example, the recognition of metastatic dural disease will have radical implications for a patient’s prognosis, as well as affording a higher chance of preservation of neurological function and quality of life with earlier appropriate treatment [3].

### Meningiomas

Meningiomas are a relatively common finding on brain imaging, with a prevalence of 53 per 100,000 people [4]. The World Health Organisation (WHO) classifies them based on their histological characteristics and recurrence risk as grade I, benign (80%), grade II, atypical (18%) and grade III, anaplastic/malignant (2%) [5, 6]. Recurrence rates are between 7–25%, 29–52% and 50–94% respectively [7]. Most arise sporadically, although some are familial or arise after radiotherapy. One notable association is that of multiple meningiomas arising in patients with neurofibromatosis type 2 (NF2) gene mutations [8].

Their morphology is either *globose*, demonstrating a rounded body growing inwards from the dura, a wide dural base and a dural tail; or *en plaque*, growing diffusely along the dura [9]. They are derived from arachnoidal cells which are most abundant near the venous sinuses, and most commonly found in the parasagittal region, sphenoid wing, middle cranial fossa, cerebellopontine angle and olfactory groove [10].

It is difficult to predict a meningioma’s grade on imaging findings. However, as a guide, it is useful to consider the features common to typical meningiomas as well as those suggestive of grade II or III lesions.

#### Imaging features (typical, grade I)

**Fig. 1:**
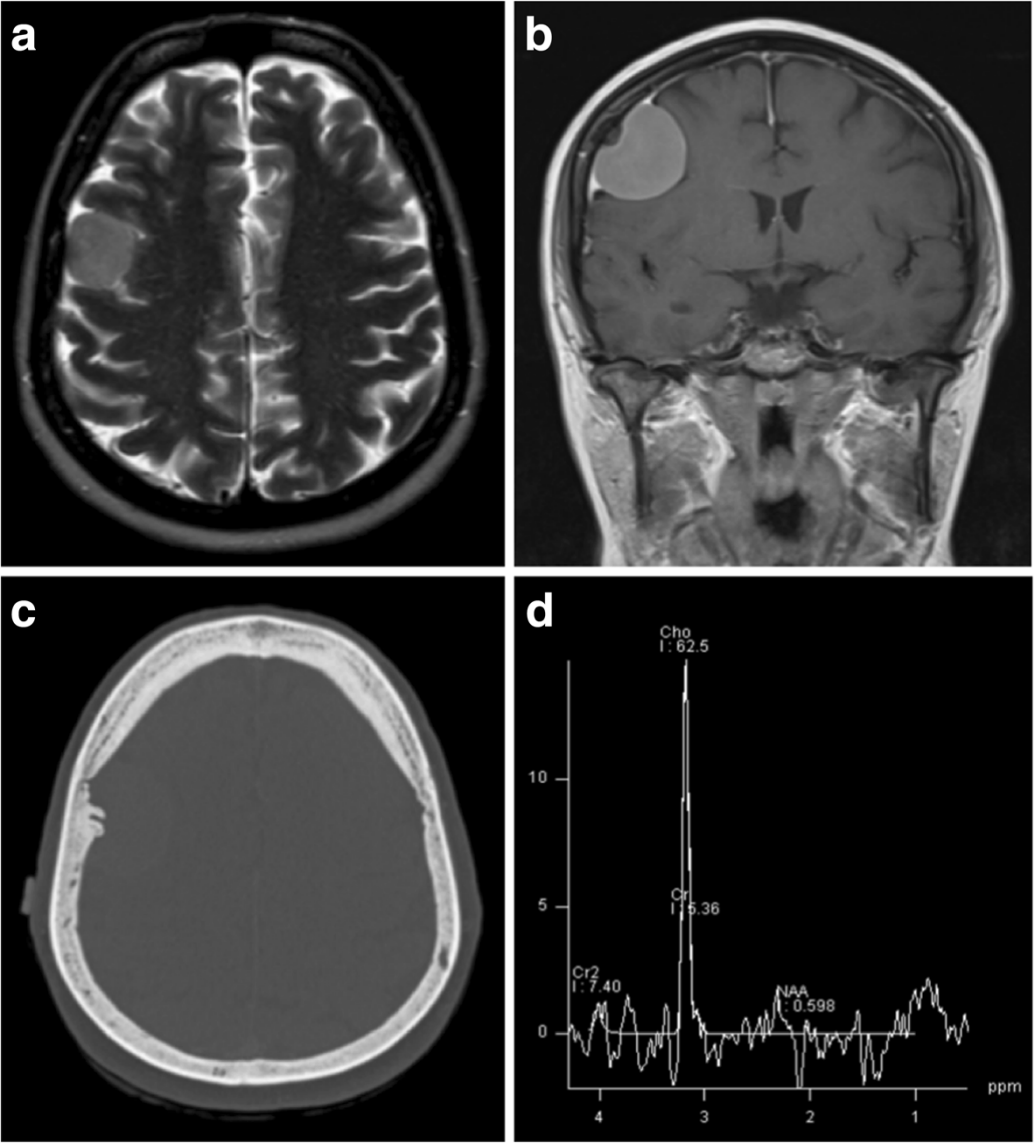
Typical meningioma. **a** Axial T2-weighted MR image of a well-circumscribed meningioma indenting the cortex with a small cleft of CSF. **b** Coronal post-contrast T1-weighted image showing uniform enhancement, an enhancing-thickened dural tail and hyperostosis of the overlying calvarium. **c** Unenhanced CT illustrating the adjacent hyperostosis of the skull. **d** Magnetic resonance spectroscopy of a similar meningioma demonstrating a high choline:creatine (Cho:Cr) ratio and a low N-acetylaspartate (NAA) peak

On computed tomography (CT) imaging, 60% of meningiomas are hyperdense to cortex, with the remainder being more isodense [11]. Up to 25% contain calcification, which is associated with slow growth and lower grade [12]. Adjacent bone exhibits hyperostosis in 20% of cases (Fig. 1c), but a small number of all grades have been associated with osteolysis [11].

Meningiomas are usually isointense to cortex on all magnetic resonance imaging (MRI) sequences (Fig. 1a) [9], and over half cause perilesional vasogenic oedema [13]. A cerebrospinal fluid (CSF) cleft is often seen between the tumour body and the brain parenchyma, and may contain displaced vessels. CSF cysts also arise in this space, which may become proteinaceous and unsuppressed on fluid suppressing sequences [9]. Where present, calcification is reflected by signal loss on gradient echo (GRE) and susceptibility-weighted images (SWI).

They almost always demonstrate uniform strong enhancement on post-contrast imaging and a dural tail sign is seen in up to 72% of cases, due to reactive thickening and enhancement of the dura (Fig. 1b) [11]. This is useful in some settings; for example, to differentiate cerebellopontine meningiomas from schwannomas, which do not typically have a dural tail. However, it is not pathognomonic and occurs in many lesions which mimic meningiomas [14].

A dural vessel may be identified on angiographic studies, often supplied by the external coronary artery (ECA) and, to a lesser extent, the internal carotid (ICA) and vertebral arteries [10]. Meningiomas envelop vessels and cranial nerves, and grow through foramina, causing nerve palsies [9]. While they seldom cause arterial insufficiency, invasion and occlusion of venous sinuses is much more common.

Meningiomas are highly vascular lesions and therefore demonstrate hyperperfusion on perfusion-weighted imaging (PWI), of which dynamic susceptibility contrast (DSC) MRI is the most commonly used. Relative cerebral blood volume (rCBV) should be raised and values are typically reported between 6 and 9 with slightly higher or lower values for angiomatous or fibrous subtypes respectively [15]. This is in contrast with metastases which have much lower rCBV values of 2 or less; the exception being the more vascular metastases of renal carcinoma or melanoma which may be indistinguishable from meningiomas [16]. Furthermore, extra-axial tumours in general can be differentiated from intra-axial tumours due to the tendency of the former’s time-intensity curve to not return to baseline during the first pass [17]. This is due to their complete lack of a blood-brain barrier allowing more vascular leakage of contrast.

On magnetic resonance spectrography (MRS), meningiomas have high choline and alanine peaks with low N-acetylaspartate (NAA) (Fig. 1d) [18]. Their higher alanine:creatine (Cr) ratios have been shown to be useful in distinguishing them from other intracranial tumours [19].

#### Imaging features (atypical, grade II)

**Fig. 2:**
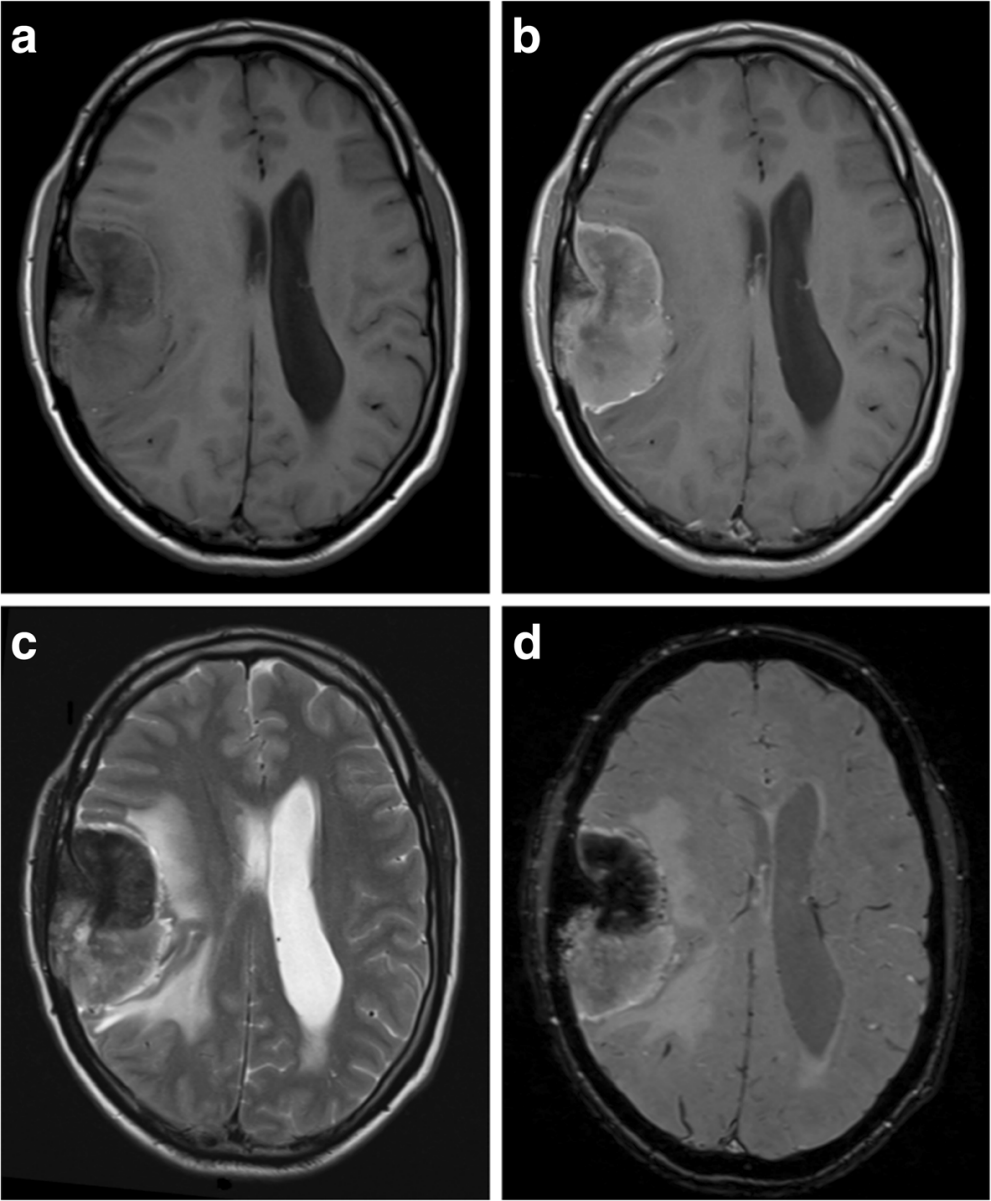
Atypical meningioma. **a**, **b** Pre- and post-contrast axial T1-weighted MR images showing a heterogeneously enhancing mass with a wide dural base, CSF cleft and bony involvement. The cortical interface is less distinct when compared with Fig. 1, and there is a moderate amount of associated vasogenic oedema. **c** Axial T2-weighted MR image of the same lesion. **d** Susceptibility-weighted images show internal calcification

Atypical meningiomas are usually located in the calvarium rather than the skull base. They commonly invade overlying bone and scalp (Fig. 2). In contrast to typical meningiomas, they have little or no calcification [20].

**Fig. 3:**
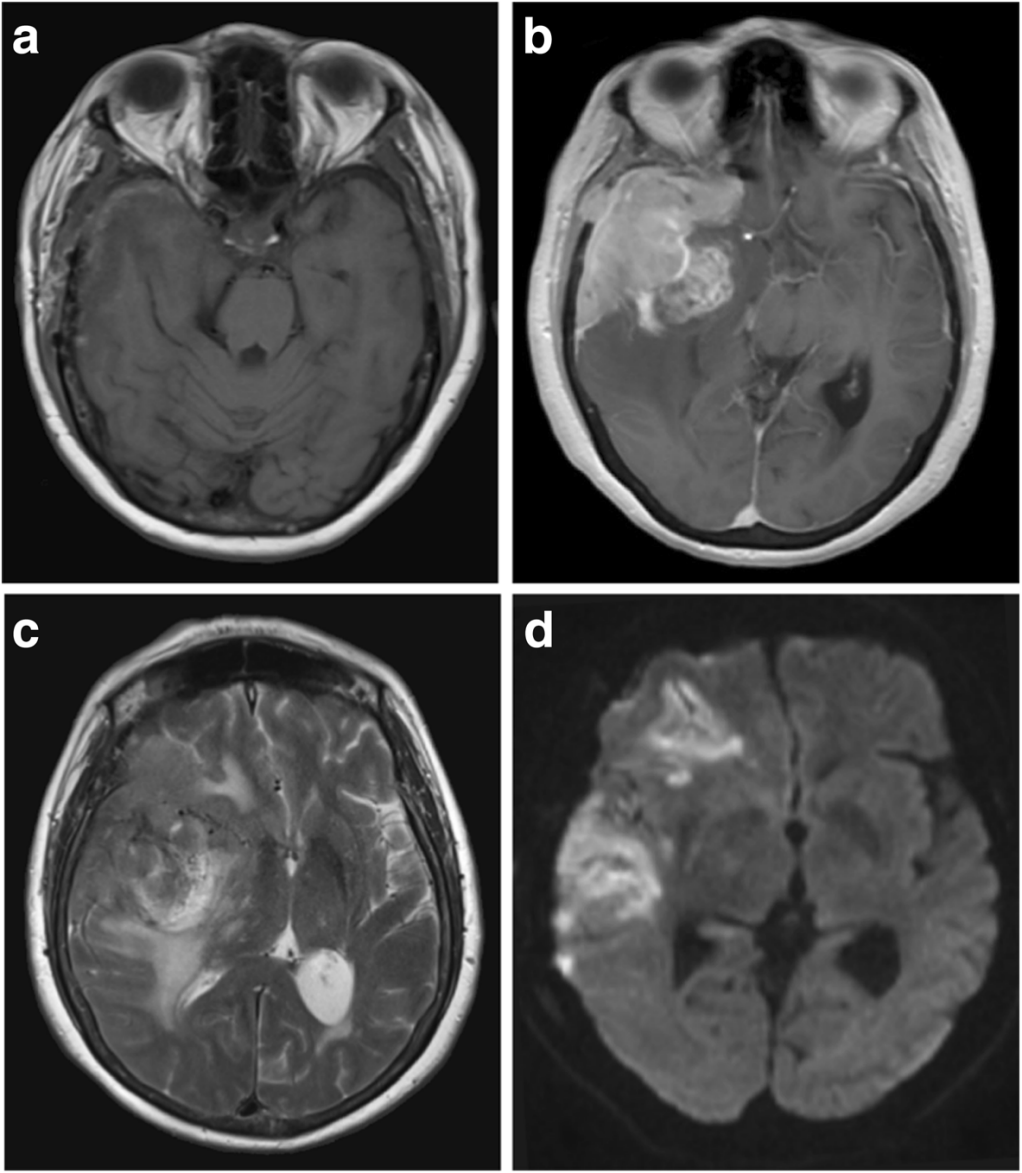
Atypical meningioma. **a**, **b** Pre- and post-contrast axial T1-weighted MR images showing a heterogeneously enhancing mass with a wide dural bass, dural tail and CSF cleft. The cortical interface is poorly outlined suggestive invasion of the underlying brain parenchyma as well as the adjacent skull. **c** Axial T2-weighted MR image of the same lesion with surrounding vasogenic oedema and midline shift. **d** A diffusion-weighted image shows heterogeneous areas of restricted diffusion extending into the adjacent cortex

Grade II lesions are characterised by microscopic invasion of brain parenchyma, displaying a less distinct interface with the adjacent cortex (Fig. 3) [21]. Contrast enhancement is more heterogeneous, and there may be areas of necrosis which do not enhance [10].

Higher-grade meningiomas, including atypical and malignant types, have lower apparent diffusion coefficient (ADC) values on diffusion-weighted imaging (DWI) (Fig. 3d); however, there is some overlap with benign types which makes assessment in individual cases difficult [22]. MRS has not been proven reliable in differentiating between typical and atypical meningiomas, although higher grades of meningioma may be associated with higher lipid and lactate peaks [18].

#### Imaging features (malignant, grade III)

Grade III lesions display features of aggressive disease, with complete loss of the CSF cleft, no demarcation between tumour and brain parenchyma, and invasion of surrounding structures [11]. They characteristically mushroom into the brain from their dural attachment. Metastases are very rare (1 in 100,000), but most arise in the lungs [23].

While intratumoural rCBV and relative mean time to enhance (rMTE) values on DSC perfusion MRI are similar between benign and malignant meningiomas, both values in the perilesional oedema of malignant subtypes can be increased due to local infiltration of tumour cells [24].

Fluorine-18 (^18^F) fluorodeoxyglucose (FDG) positron emission tomography (PET) imaging has been found to be useful for predicting risk of recurrence along with meningioma grade [25]. Lesions with good surgical outcomes had the least amounts of tracer uptake, with atypical and recurrent meningiomas demonstrating higher metabolism [26]. Other tracers such as 11C-choline and 11C-methionine have higher sensitivity compared with FDG for detecting lesions due to lack of adjacent grey matter uptake and perilesional uptake reveals brain invasion not detected on anatomical MRI sequences [27].

### Dural metastasis

Dural metastases occur by one of four main mechanisms: direct extension from overlying skull (61%); haematogenous spread (33%); and to a lesser extent, lymphatic spread or retrograde spread via the vertebral venous plexus [28]. A small number also result from intraoperative seeding. They are a common, being found in up to 8–9% of cancer patients at post-mortem [28]. Most originate from cancers of the breast (34%), prostate (17%) and lung (13%), although others including bowel and germ cell tumours have been described [29]. Other intracranial deposits are usually present, with only 4% of patients having isolated dural intracranial disease [28]. The incidence of intracranial metastasis is currently increasing due to improving treatments for systemic cancer [30].

Intradural metastases remain outside the blood-brain barrier which allows penetration of chemotherapy [28]. This is different to parenchymal and leptomeningeal deposits where the blood-brain barrier impedes its action. Outcomes are therefore relatively good for dural lesions, and the distinction should be made when imaging is used to plan therapy regimens.

#### Imaging features

**Fig. 4:**
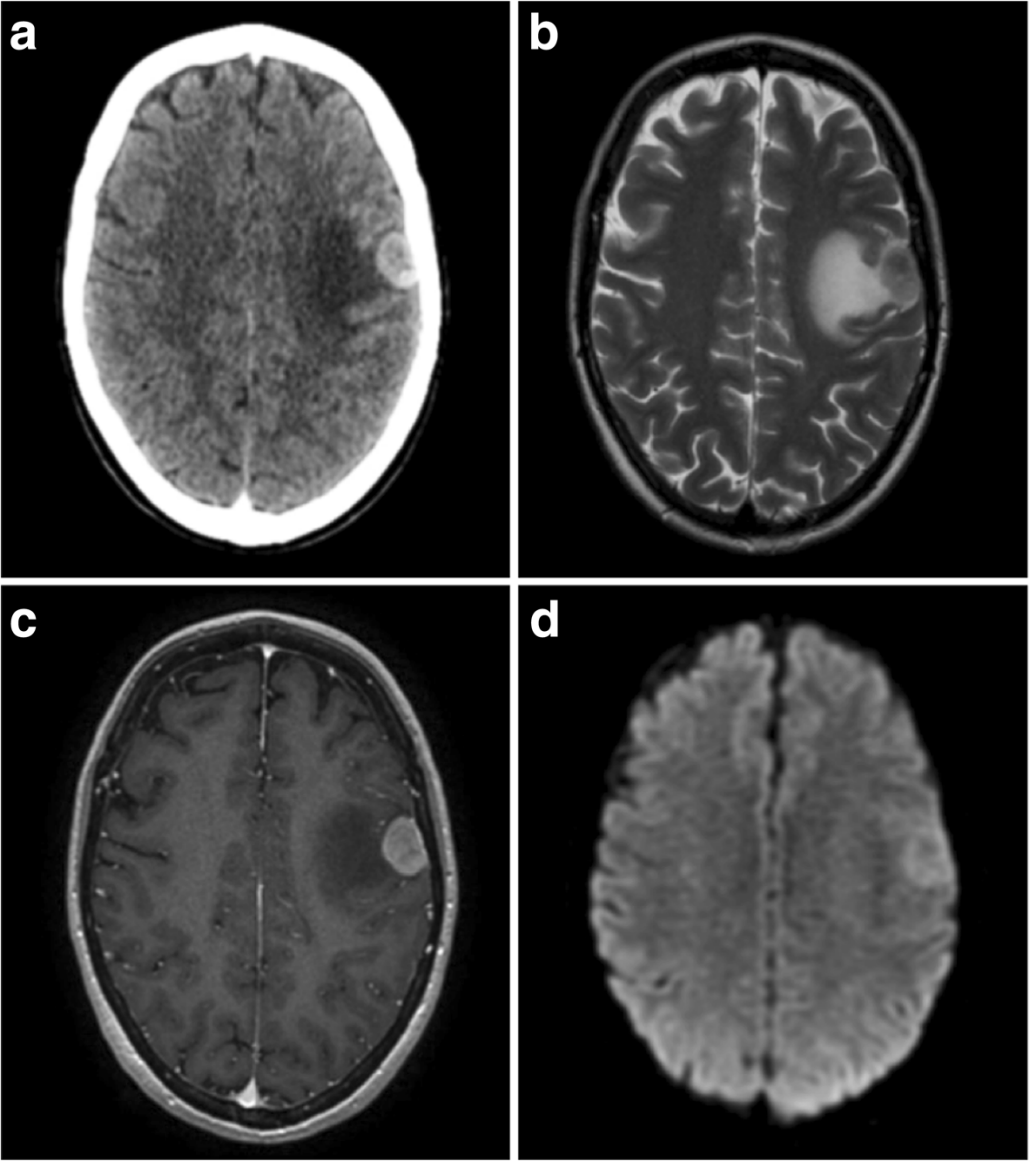
Breast cancer metastasis. **a** Post-contrast axial CT showing a strongly enhancing extra-axial lesion with a wide dural base and associated vasogenic oedema. There is no bony hyperostosis or erosion. **b** T2-weighted axial image of the same lesion which has an indistinct cortical interface suggestive of involvement. **c** Post-contrast axial T1-weighted MR image of the lesion with some slight heterogeneous but strong enhancement. **d** Diffusion-weighted imaging demonstrated intermediate restricted diffusion within the lesion

**Fig. 5:**
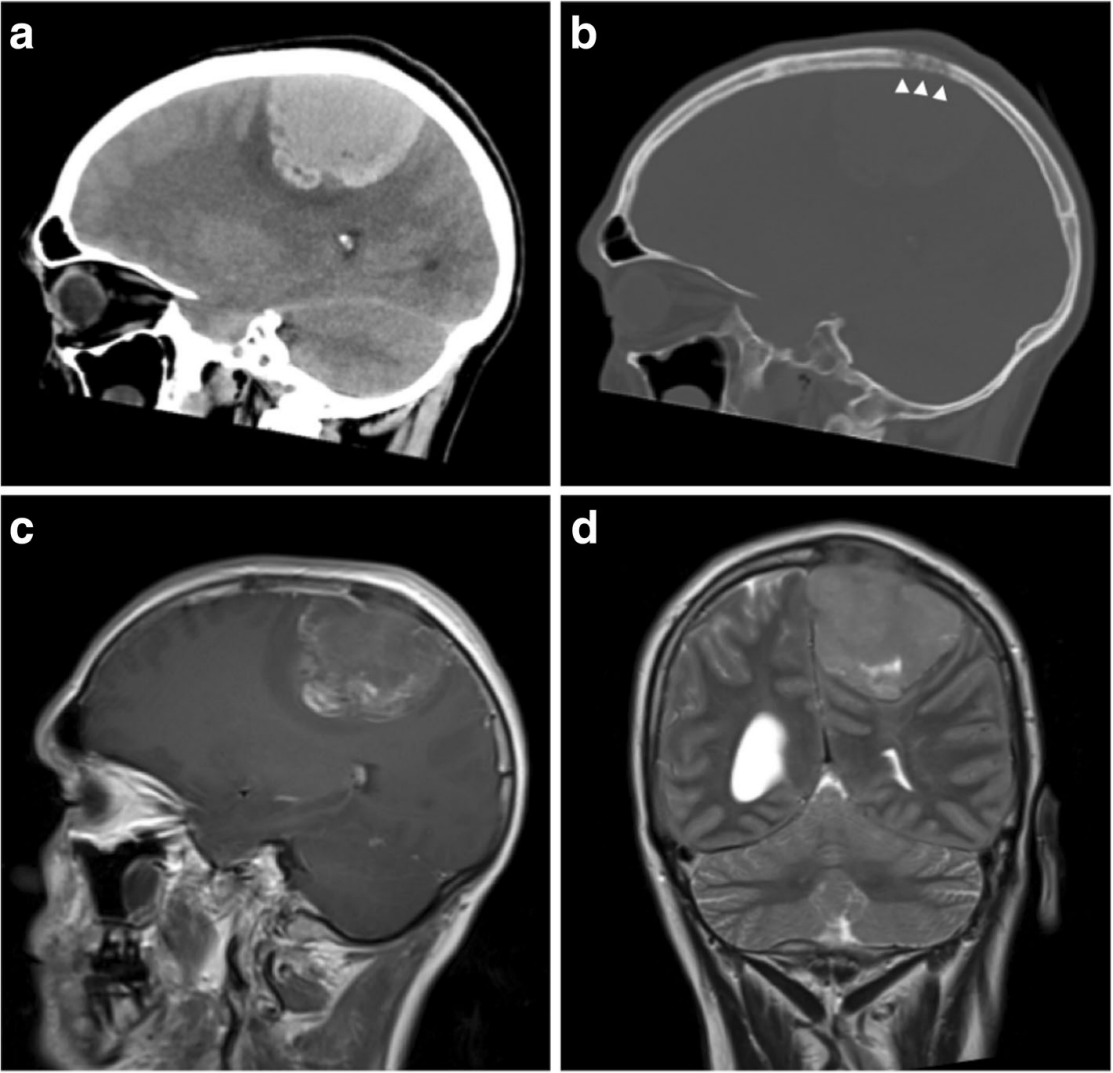
Non-germ cell seminoma metastasis. **a** Sagittal unenhanced CT image showing an irregular and hyperdense extra-axial mass with a wide dural attachment and only minimal vasogenic oedema. **b** Sagittal CT image on the bone windowing showing bony destruction adjacent to the lesion (white arrowheads) and no hyperostosis. **c** Post-contrast sagittal T1-weighted MR image showing only minimal irregular peripheral enhancement and no dural tail. The sagittal sinus is involved and occluded. On unenhanced T1-weighted images, the lesion was isointense to white matter and contained small areas of T1-shortening consistent with haemorrhage. **d** Coronal T2-weighted image of the same lesion

**Fig. 6:**
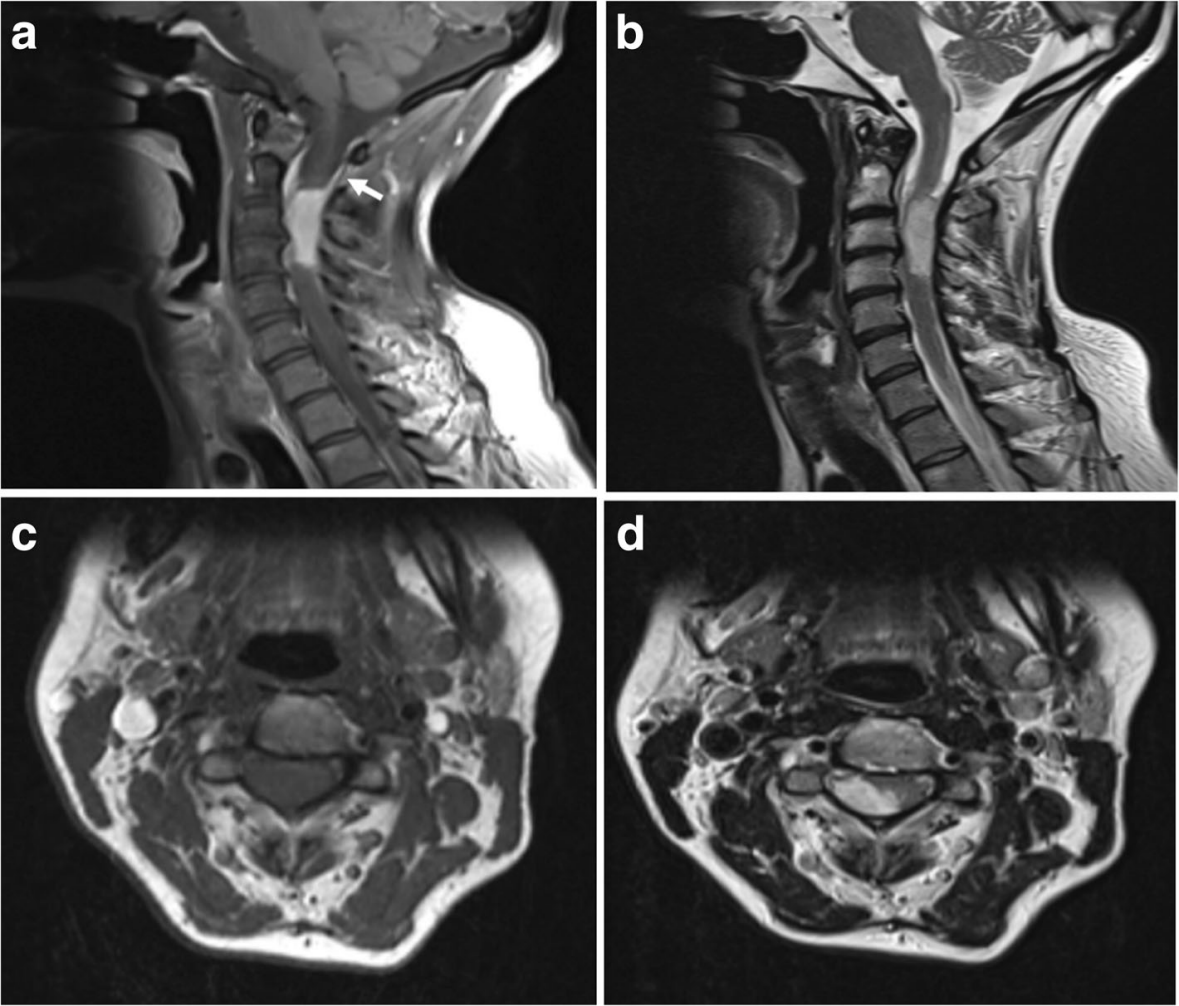
Dural glioblastoma metastasis. **a** Post-contrast sagittal T1-weighted MR image showing an avidly enhancing intra-dural, extra-medullary lesion within the upper posterior cervical spine. An enhancing dural tail is seen superiorly (white arrow). **b** T2-weighted sagittal images of the same lesion. **c**, **d** Axial T1 and T2-weighted images show the posterior intra-dural and extra-medullary location of the lesion which is compressing the cord to the left

On imaging, they appear as focal nodular thickening of the dura, or when there is diffuse involvement, as smooth dural thickening following the contour of the calvarium [28]. They can be associated with vasogenic oedema (Fig. 4b) and, if large, they indent the cortex. They enhance strongly (Figs. 4, 5 and 6) and, like meningiomas, an enhancing dural tail is present in nearly half of cases [28]. In some instances, they are associated with spontaneous subdural [3], or rarely parenchymal haemorrhage [31], which may obscure the underling lesion.

On CT images, they are hyperdense to cortex and seldom undergo calcification [32]. Soft tissue metastases commonly invade and cause destruction of the adjacent skull (Fig. 5b), although prostate cancer with osteoblastic bony involvement can mimic the hyperostosis of meningiomas [3].

Lesions are usually isointense or hypointense on T1-weighted MR images (T1WI), but are variable on T2-weighted MR images (T2WI), sometimes being predominantly hyper- or hypointense. Direct invasion of brain parenchyma with signal change is observed in around a third of patients (Fig. 4b) [28]. Lesions tend to demonstrate facilitated diffusion on DWI [32].

Most dural metastases exhibit reduced perfusion when compared with meningiomas, with typical rCBV values of less than 2 [16]. The exceptions are renal carcinoma, melanoma and Merkel cell neuroendocrine skin carcinoma metastases which are hypervascular and may be indistinguishable on perfusion imaging. Hypervascular metastases such as these are often hyperdense on CT due to intralesional haemorrhage which may aid diagnosis [32]. Melanoma metastases are typically hyperintense on T1WI, in contrast with meningiomas. On dynamic perfusion imaging, metastases have also been shown to have lower relative wash-in times than meningiomas [33].

On MRS, metastases have low NAA:creatine ratios and high lipid:creatine ratios without the alanine peak characteristic of meningiomas [32].

Rarely, the phenomenon of tumour-to-tumour metastasis occurs, with development of a metastatic deposit within an established meningioma [34]. Most described cases involve spread of lung or breast cancer to a low-grade meningioma.

### Solitary fibrous tumours/haemangiopericytomas

Until recently, haemangiopericytomas were classified separately from solitary fibrous tumours (SFT) by the WHO, but due to histological overlap, they are now considered a higher grade of SFT [5, 35]. The traditional SFT, collagenous, containing spindle cells and of low cellularity, is now grade I SFT; haemangiopericytomas, with low collagen, plump cells and stag-horn vasculature, are now classified as grade II; highly mitotic lesions (previously anaplastic haemangiopericytomas) are classified as grade III [5]. However, debate is still ongoing in neuropathology literature about whether they should be formally separated, in part due to their distinct clinical outcomes [36].

SFT are rare mesenchymal tumours that more often occur in the mediastinum, abdomen and skin [37]. Occasionally, they arise intracranially, almost always from the meninges [37]. Currently, the only definitive management is surgery [38]. Low-grade tumours are often well circumscribed and resection is usually performed initially; however, adjuvant radiotherapy is used if these lesions are incompletely removed.

As tumours may be large and hypervascular, pre-operative embolisation may need to be considered [39]. Follow-up imaging is essential to assess for recurrence, particularly in cases of difficult resection or unusual histology. In higher-grade tumours, recurrence is extremely common, occurring in up to 94% at 15 years [40]. Distant metastases occur in approximately 15% of cases [41], most spreading to the lungs, bone and liver [40].

#### Imaging features (grade I)

**Fig. 7:**
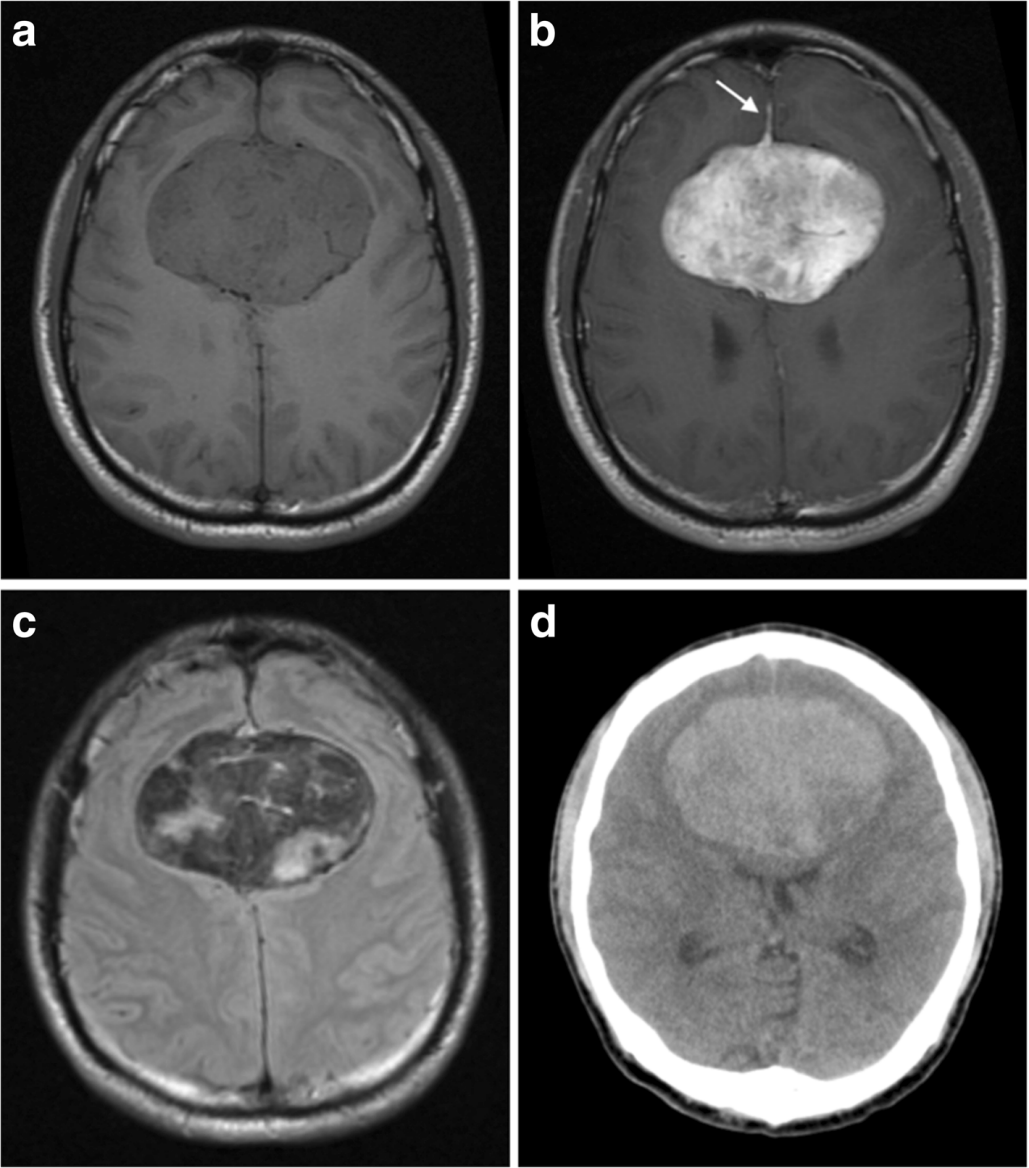
Solitary fibrous tumour. **a**, **b** Pre- and post-contrast axial T1-weighted MR images showing an avidly enhancing extra-axial lesion straddling the falx cerebri. An enhancing dural tail is seen anteriorly (white arrow). **c** On T2 FLAIR images, the lesion has regions of high and low signal consistent with its fibrous collagen composition. Note how these low signal regions enhance with administration of contrast in Fig. 7b. **d** On unenhanced CT images, the mass is largely hyperdense with no areas of calcification

Intracranial SFTs almost always occur extra-axially, are often lobulated and have a dural attachment (Fig. 7) [37]. Location is not a particularly helpful discriminating factor as tumours occur in similar places to meningiomas.

On CT, they are isodense to hyperdense to cortex (Fig. 7d). Calcification is uncommon, and smooth erosion of the overlying skull is seen in around 57% [37]. There is no overt destruction of overlying skull in low-grade lesions.

On MR, lesions are of homogeneous and isointense signal on T1WI and nearly always heterogeneous with low signal on T2WI (Fig. 7c) [37]. Even areas of low fluid attenuated inversion recovery (FLAIR)/T2 signal tend to enhance strongly, a characteristic that helps distinguish them from meningiomas (Fig. 7b). A small proportion also display a dural tail sign (Fig. 7b). Regions of collagen appear as either curvilinear streaks or large regions of hypointense signal on T1WI and T2WI [42]. In the latter case, these appearances give rise to an appearance described in the literature as a ‘Yin-Yang’ pattern. This is due to half the lesion being hyperintense and the other half being hypointense. Two thirds of lesions contain areas of restricted diffusion [37].

On digital subtraction angiography, there is a strong tumour blush with a pial and meningeal supply [37]. In contrast to meningiomas, low-grade SFTs do not invade or occlude nearby venous sinuses.

Solid regions of tumour demonstrate restricted diffusion on DWI [37]. On MRS, there are lipid and lactate peaks with high myo-inositol peaks also observed, which can help differentiate them from meningiomas [37].

On perfusion imaging, SFTs are hyperperfused with rCBV values of 7 to 7.5 [43]. While these values are similar to those reported for meningiomas, this can help differentiate SFTs from other extra-axial lesions such as metastases.

#### Imaging features (grade II–III)

Higher-grade lesions have similar imaging appearances to atypical meningiomas [44, 45]. They are more likely to mushroom towards the brain parenchyma due to a narrow dural base and have a lobulated irregular outline. Grade III lesions are also more likely to cross the midline and invade adjacent dural venous sinuses, as well as overlying skull and soft tissues [44].

On CT, masses are hyperdense but heterogeneous, with strong enhancement of solid components [44]. Mixed signal intensity is seen on all MR sequences with areas of necrosis, cystic degeneration and haemorrhage seen especially in grade III lesions, with multiple flow voids seen due to high vascularity [46]. Enhancement is also more heterogeneous with increasing grade. An associated dural tail is common but less so in grade III lesions likely due to rapid growth [44]. SWI typically reveals multiple internal flow voids [47]. As in low-grade lesions, MRS shows high myo-inositol [37, 48].

High-grade SFTs have been reported to have only low-grade tracer uptake on FDG-PET studies, [49] with minimal tracer uptake seen even in metastatic high-grade SFT deposits [50].

### Lymphoma

Central nervous system (CNS) lymphoma is classified as *primary*, when diagnosed in the absence of systemic disease; or *secondary*, as an extranodal feature of systemic lymphoma. Primary CNS lymphoma (PCNSL) is rare and accounts for 1% of all extranodal lymphoma cases, and 0.6% of intracranial tumours [51]. Nearly all primary disease is diffuse large B-cell lymphoma or high-grade Burkitt-like B-cell lymphoma [52]. While rare in the general population, it is relatively common in immunocompromised patients.

Primary dural lymphoma makes up a small proportion and is usually low-grade, B-cell marginal zone lymphoma [53], a comparatively indolent subtype more common in gastrointestinal tract where it is known as mucosa associated lymphoid tissue (MALT) lymphoma. This distinction is important as most other forms of primary CNS lymphoma have different therapeutic regimens and a poorer prognosis. There is however a risk of transformation from MALT lymphoma to higher-grade diffuse B-cell lymphomas [54].

Secondary CNS lymphoma refers to either CNS involvement of systemic disease or an isolated recurrence. The frequency is variable depending on the histological subtype, being low in Hodgkin lymphoma (up to 0.5%) [55] but as high as 27% in non-Hodgkin lymphoma [56]. Unlike primary disease, it more commonly affects the leptomeninges. This is often not evident radiologically and is diagnosed more readily with CSF cytology.

#### Imaging features

**Fig. 8:**
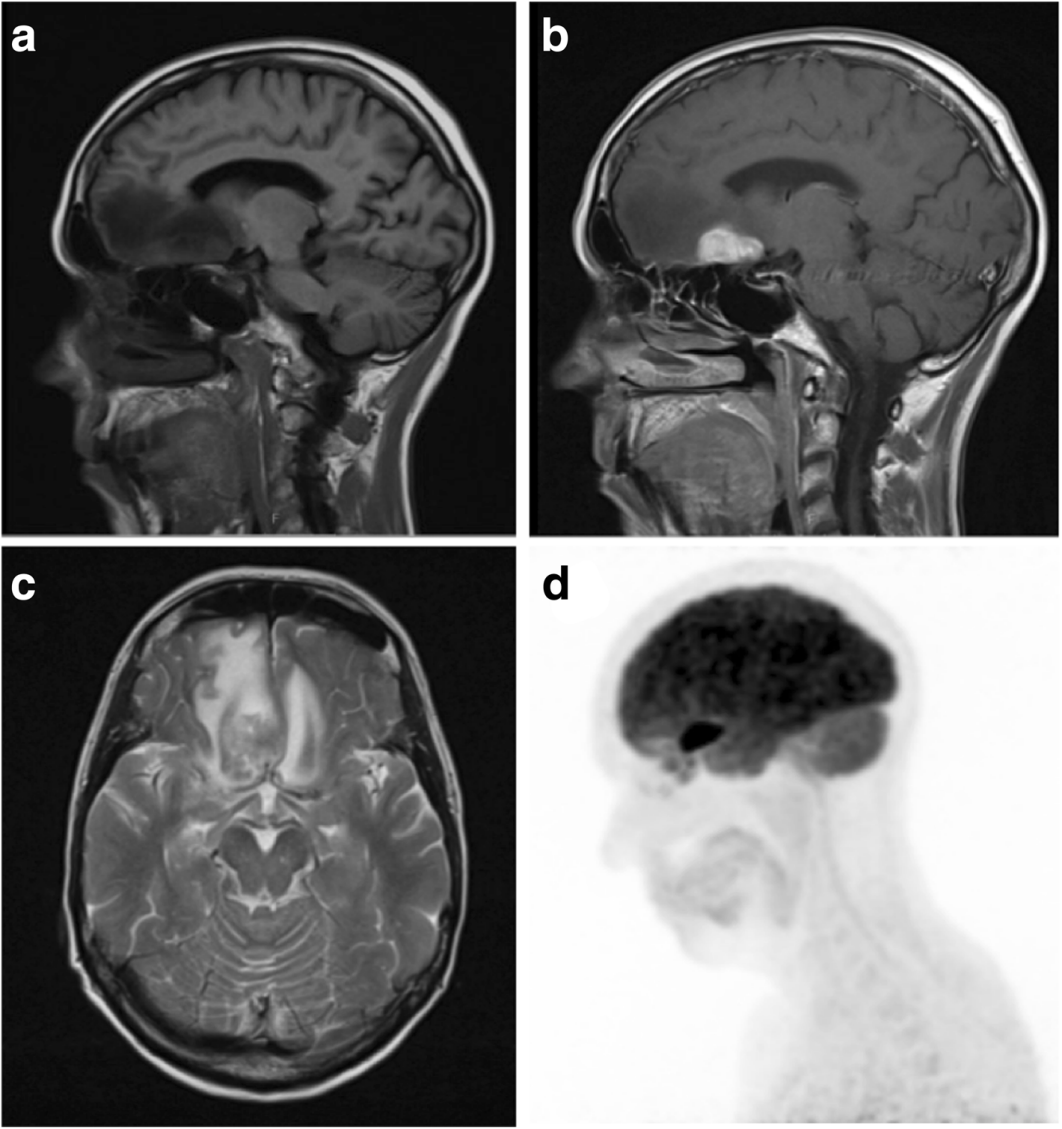
Lymphomatous deposit. **a**, **b** Pre- and post-contrast sagittal T1-weighted MR images showing a solitary extra-axial lymphomatous deposit with a wide dural base and avid enhancement. It is lobulated, and its border is ‘fluffy’ and less distinct than that of the typical meningioma. There was no skull hyperostosis or intratumoural calcification on CT imaging. **c** Axial T2-weighted MR image of the same lesion showing marked vasogenic oedema. **d** Maximum intensity projected FDG PET image demonstrates avid tracer uptake within the lesion

**Fig. 9:**
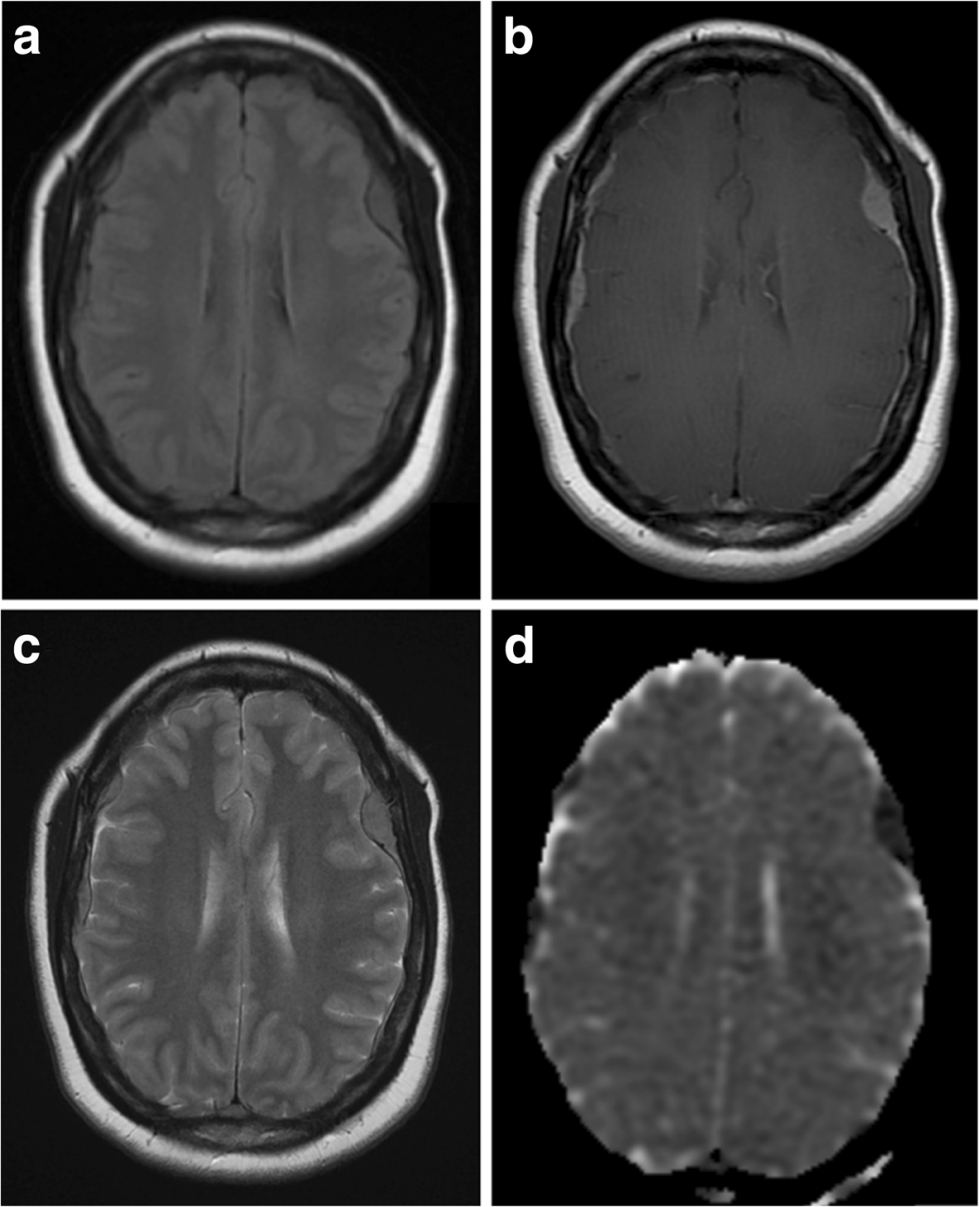
Multifocal dural lymphoma. **a**, **b** Pre- and post-contrast axial T1-weighted MR images showing diffuse dural thickening and enhancement with superimposed dural masses. **c** Axial T2-weighted MR image of the same lesions. **d** Apparent diffusion coefficient (ADC) map showing intense restricted diffusion within the multiple dural mass lesions in keeping with high cellularity

Common locations for primary dural lymphoma include the falx cerebri, tentorium cerebelli and parasellar regions [57], and lesions are more commonly associated with more vasogenic oedema than meningiomas (Fig. 8c) [58]. Up to 50% of patients have more than one mass at the time of imaging (Fig. 9). Diffuse dural lymphoma can mimic en plaque meningioma or even subdural haematomas due to being hyperdense on CT images [59, 60].

These highly cellular masses tend to be hyperdense on CT, hypointense on T1WI and iso- to hypointense on T2WI, demonstrating strong uniform enhancement (Figs. 8b and 9b). They may also have a dural tail and be associated with both hyperostosis and bony erosion. Intralesional calcification or haemorrhage is rare, and not usually seen, even on SWI [57]. They are typically lobulated and have a ‘fuzzy’, indistinct brain-tumour interface (Fig. 8b) [58]. Due to their high cellularity, lymphomatous lesions demonstrate restricted diffusion on DWI, more so than meningiomas and metastases [61]. Raised lipid/lactate peaks with high Cho:creatine ratios are seen on MRS [57, 62]. While these characteristics are also found in metastases and glioblastomas, they can help in differentiating from other lesions such as meningioma [61]. On PWI, PCNSL lesions typically have lower rCBV than other intracranial tumours including meningiomas [15, 57, 63].

Lesions are metabolically active and show avid tracer uptake on FDG-PET (Fig. 8d). This has been shown to be useful in distinguishing lymphomatous lesions from meningiomas, which tend to have low activity [64], as well as for monitoring treatment response. Carbon-11 (^11^C) methionine PET has also been utilised and shows a larger area of involvement than standard contrast-enhanced MRI, suggesting it is a more sensitive tool, particularly in detecting more subtle leptomeningeal disease or in evaluating disease response or recurrence [65].

In immunocompetent patients, PCNSL has been shown to retain high levels of iodine 123 N-isopropyl-p-iodoamphetamine tracer using single-photon emission computerised tomography (SPECT) imaging [57]. Alternatively, in immunocompromised patients, PCNSL lesions may be differentiated from infection-related lesions, due to the former demonstrating increased thallium 201 tracer uptake ratios on SPECT, as well as increased FDG tracer uptake on PET imaging.

### Melanocytic neoplasms

Melanocytes are a normal cell type found within the leptomeninges, mostly around the base of the brain, brainstem and anterior cervical cord, where they are found in similar proportions to those in cutaneous tissue [66]. Primary melanocytic tumours of the CNS are rare, with most lesions being metastatic. However, primary dural disease does occur, and it represents a varied spectrum which includes primary melanoma, meningeal melanocytoma, neurocutaneous melanosis and primary melanosis [65].

It is essential to differentiate primary malignant melanoma from metastatic meningeal spread as this alters the prognosis and therapeutic approach considerably. The absence of a known primary melanoma outside the neuroaxis and absence of any accompanying parenchymal lesions make primary disease more likely, although systemic disease should be excluded in the first instance. Ninety percent of metastatic melanoma is cutaneous in origin, and review of the patient history or examination aids diagnosis in most cases [66].

#### Imaging features

Due to the distribution of melanocytes in the leptomeninges, primary lesions most commonly arise around the skull base and brainstem, with supratentorial disease being rare [67]. On CT images, melanocytomas appear as isodense or hyperdense to cortex and enhance strongly after contrast administration, appearing similar to meningiomas [68]. In contrast to meningiomas, they seldom demonstrate calcification or hyperostosis.

Melanin typically causes T1 shortening on MR imaging, and lesions are generally hyperintense due to their high-melanin content [69]. The degree of hyperintensity within the lesion will also be contributed to by any areas of haemorrhage which often occur in melanocytic tumours. On T2WI, these lesions are usually isointense or hypointense. Lesions demonstrate central diffusion restriction [70]. The appearance of primary and secondary meningeal melanoma is very similar to this, although the enhancement pattern may be heterogeneous, nodular or peripheral, rather than homogeneous [71]. Melanoma CNS metastases, when occurring peripherally, particularly around the cerebellopontine junction and internal auditory canal, mimic the MR appearances of meningiomas, often being isointense on T1WI and slightly hyperintense on T2WI [71].

Dural melanocytic lesions have been reported as having increased choline and lactate as well as low NAA and creatine on MRS [70]. Choline:creatine and choline:NAA ratios are also increased. Similar to SFTs, increased myo-inositol has been described [70].

99m Tc-sestamibi (MIBI) SPECT and 99m Tc-hexamethylproyleneamine ox-ime (HMPAO) SPECT have been shown to be of use due to increased uptake in primary melanocytic tumours [72–74].

### Gliosarcoma

Gliosarcoma is a rare primarily glial tumour, accounting for 1–8% of glioblastomas and < 0.5% of intracranial tumours [75]. Histologically, they demonstrate both glial and sarcomatous differentiation [75]. Their pathogenesis remains under debate with some suggesting the sarcoma component arises from the adventitia of blood vessels, with islands of glioma cells; and others proposing they have a monoclonal origin, with aberrant mesenchymal differentiation of glial cells forming the sarcoma component [76].

When they occur peripherally in the brain, they may invade the meninges and develop into a desmoplastic encapsulated mass which appears radiologically and macroscopically like a meningioma [75]. They carry a poor prognosis with a high rate of recurrence.

#### Imaging features

**Fig. 10:**
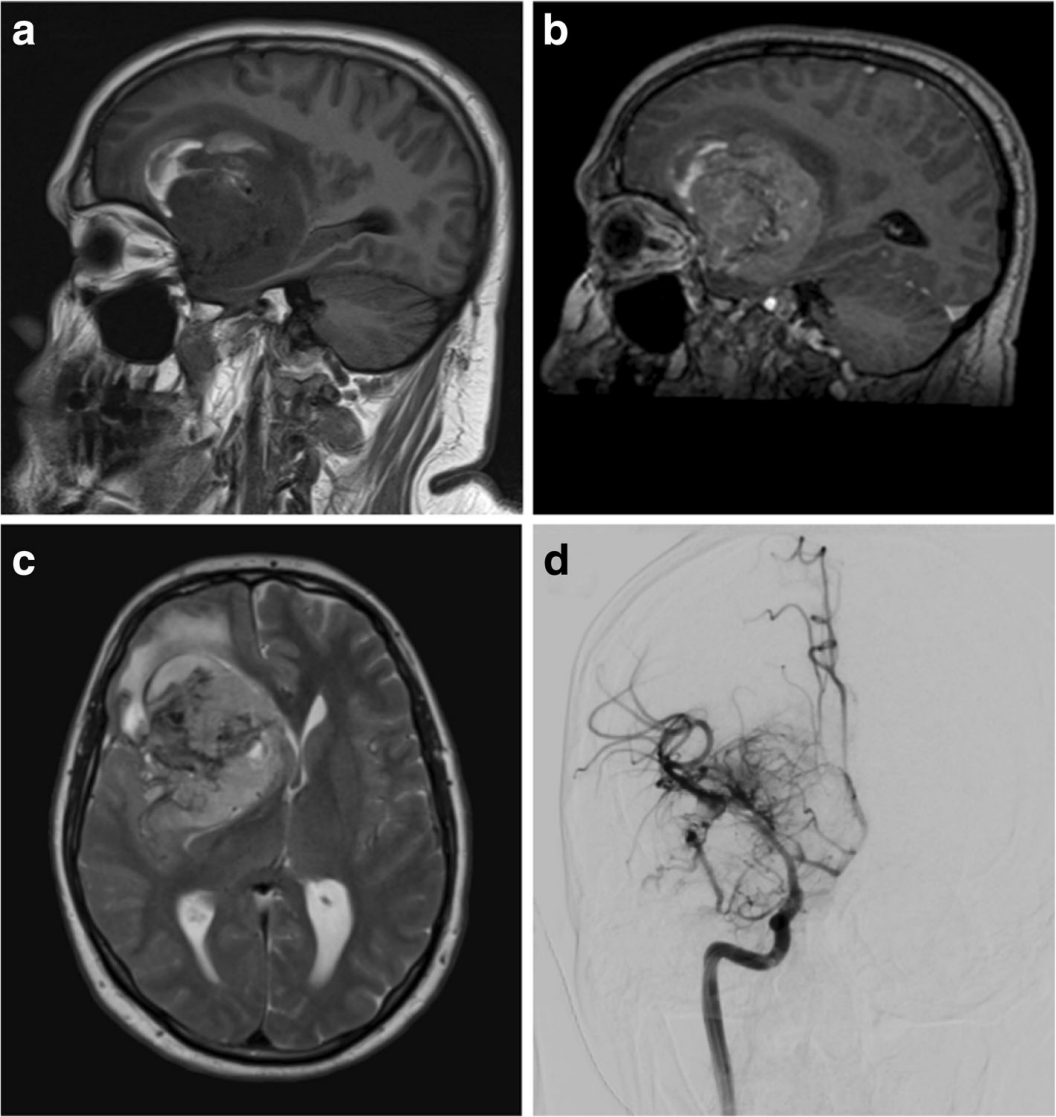
Glioblastoma. **a**, **b** Pre- and post-contrast sagittal T1-weighted MR images showing a vascular and heterogeneously enhancing lesion with areas of internal necrosis and haemorrhage. **c** T2-weighed axial MR image of the same lesion showing internal flow voids, an apparent thin CSF cleft and a small amount of perilesional oedema. **d** Selective right internal carotid artery angiogram demonstrates a highly vascular mass with multiple abnormal and aneurysmal vessels (performed pre-embolisation)

Two types of radiological appearance are seen in gliosarcomas: deep parenchymal lesions often have an ependymal attachment and are more often accompanied by satellite lesions. Peripherally located lesions however are often solitary and associated with a dural attachment simulating meningioma (Fig. 10) [75].

On CT images, lesions are predominantly hypodense but have hyperdense soft tissue components and may contain areas of haemorrhage [77]. They do not form calcification and do not cause hyperostosis of overlying bone. On T1WI, lesions are generally hypointense with surrounding isointense regions. Scattered hyperintensity is seen with areas of haemorrhage. On T2WI, lesions are heterogeneous but generally have central hyperintensity with surrounding isointensity. Around 44% of tumours are homogenous, well demarcated and demonstrate strong homogenous enhancement, with a quarter of all lesions having a dural tail sign [75]. The rest are heterogeneous, but only a third have the peripheral ring-enhancement characteristic of glioblastomas. A small proportion (6%) also contains areas of haemorrhage, but calcification is uncommon (Fig. 10a).

Intralesional diffusion restriction is heterogeneous and ranges from mild to strong restriction [77]. On MRS, solid enhancing parts of the tumours show lactate peaks, along with increased choline, low NAA and low creatine values [77]. Particularly high lactate peaks are seen in central cystic areas. Peritumoural regions may also demonstrate areas of increased Cho:Cr and decreased NAA:Cr ratios [77].

Gliosarcomas are hypermetabolic with high FDG tracer uptake within solid tumour portions and areas of photopenia which relate to central necrosis [78].

### Rosai-Dorfman disease

Rosai-Dorfman disease (RDD), or sinus histiocytosis with massive lymphadenopathy, is a benign idiopathic lymphoproliferative disorder. Most patients present with bilateral painless cervical lymphadenopathy, pyrexia, raised white cell count and erythrocyte sedimentation rate (ESR) with hypergammaglobulinaemia. Rarely, extranodal disease occurs affecting the respiratory tract, eyes, skin and in less than 5% of cases the CNS [79]. In contrast to systemic RDD, which usually affects children, intracranial disease occurs later at a mean age of 39 years and usually occurs in isolation [80].

In systemic RDD, treatment is only advised in symptomatic cases or where the presence of disease is impairing the function of affected organs [80], with spontaneous resolution occurring in 20% of patients [81]. However, this has never been reported in cases of intracranial RDD, and treatment is usually with complete resection, which also aids diagnosis [80]. Adjuvant radiotherapy, chemotherapy and steroid-therapy have been used, but evidence of their effectiveness is limited.

#### Imaging features

Intracranial lesions are extra-axial and almost always have a dural attachment. They arise around the parasellar, parafalcine, cavernous sinus and petroclival regions, although sometimes from the endocranium [80]. They are homogenous, well-circumscribed, iso to hyperdense on CT, without calcification and tend to erode adjacent bone with no hyperostosis [82]. A small amount of vasogenic oedema is often present in the underlying white matter.

On MR images, they are isointense on T1WI and isointense to hypointense on T2WI. They enhance strongly and homogeneously, and a dural tail sign is common [80]. Descriptions of spectroscopy findings are sparse, but lesions have been reported to demonstrate high choline peaks with low NAA and lactate [83]. When angiographic imaging is performed, lesions are seen to be most often minimally vascular with only a faint contrast blush [84]. Similarly, perfusion-weighted sequences elicit low rCBV in contrast to the high values seen in meningiomas and SFT [85].

### Erdheim-Chester disease

Erdheim-Chester disease, also known as polyostotic sclerosing histiocytosis, is a rare form of non-Langerhans histiocytosis [86]. It is a systemic process in which tissues undergo infiltration by foamy histiocytes with resulting fibrosis. The disease usually presents in the fifth decade and is slightly more common in males [87].

About half of patients have extra-skeletal disease and three types of intracranial involvement have been described [85]. In order of prevalence, these are divided into *infiltrative pattern*, with widespread intracranial parenchymal lesions; *meningeal pattern*, with nodular diffuse meningeal thickening, or discrete meningioma-like nodules; and *composite pattern*, with both features of the other two patterns [88]. Most frequently, patients present with diabetes insipidus, cerebellar dysfunction and focal deficits due to mass effect [89].

#### Imaging features

On CT, dural masses are well circumscribed and hyperdense. On MRI, they are isointense on T1WI and typically iso- or hypointense on T2WI. They enhance avidly and may have a dural tail sign [14]. If present, the associated dural tail has been shown to enhance for up to 8 days [90]. This is thought to be due to gadolinium phagocytosis by abnormal histiocytes. Additional intraparenchymal lesions are extremely rare but if present manifest as areas of high signal on T2WI within the cerebral peduncles and dentate nucleus with associated post-contrast enhancement [85].

Eighty percent of patients with head and neck involvement have diffuse facial bone or calvarial thickening. Fifty-eight percent of these having bilateral maxillary and sphenoid sinus wall osteosclerosis, and 21% have ethmoidal air cell wall osteosclerosis [89]. While best seen on CT, this is also evident as bone hypointensity on T1WI and T2WI. Lesions show avid tracer uptake on PET imaging [90].

### Tuberculosis

Despite being most prominent in developing countries, the incidence of tuberculosis is rising elsewhere, particularly in migrant populations and due to an increased prevalence of human immunodeficiency virus [91]. The infection is typically confined to the respiratory system, however can progress to multisystem disease, particularly in immunocompromised patients [92]. CNS disease occurs in around 1% and constitutes 10–15% of extra-pulmonary disease [93].

Mycobacteria reach the meninges via haematogenous spread during the primary pulmonary infection, where they establish Rich foci in the meninges, subpial or subependymal regions [94]. These foci rupture into the subarachnoid space and other intracranial compartments, resulting in suppurative basal meningitis, cerebritis or abscess formation. Parenchymal tuberculomas also directly involve the meninges or rupture to cause pachymeningitis or leptomeningitis.

#### Imaging features

**Fig. 11:**
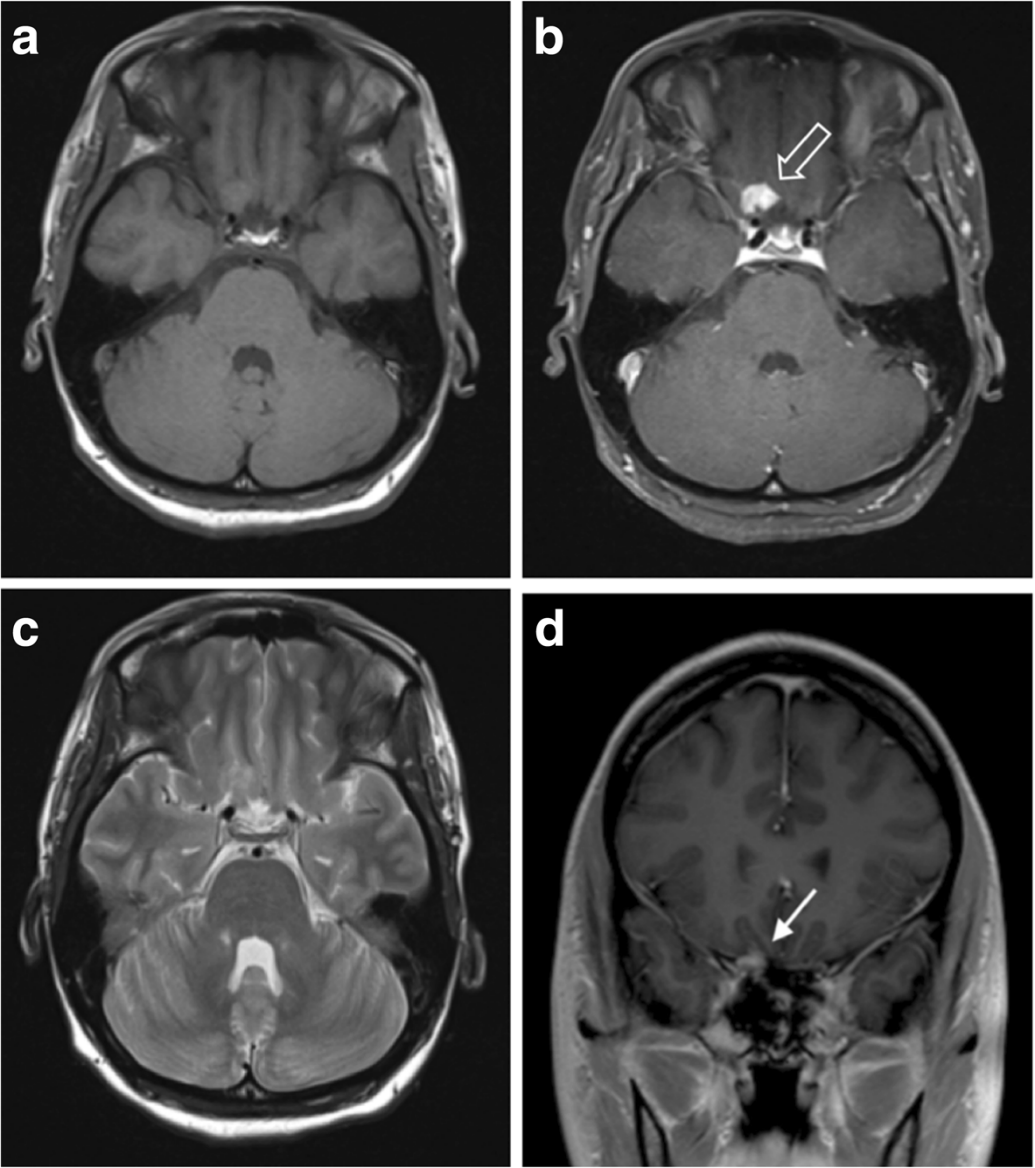
Solitary tuberculoma. **a**, **b** Pre- and post-contrast axial T1-weighted MR images showing a strongly and homogenously enhancing lesion with a wide dural base at the right orbital apex (outlined arrow). No other intracerebral or meningeal lesions were seen. **c** T2-weighed axial MR image of the same lesion showing an isointense lesion with no associated vasogenic oedema. **d** Post-contrast coronal T1-weighted MR image demonstrating the lesion’s dural attachment (solid white arrow)

Tuberculous meningitis predominantly affects the basal meninges and has two patterns: *focal nodular*, with mass-like thickening of the meninges; or en plaque, with diffuse meningeal thickening [95]. Affected areas are isodense to hyperdense on CT [93]. On MR, lesions appear isointense on T1WI and T2WI and demonstrate avid uniform enhancement (Fig. 11). They are typically associated with a greater amount of perilesional vasogenic oedema than meningiomas.

En plaque thickening may involve cranial nerves causing cranial nerve palsies. Basal exudates result in inflammatory vasculitis leading to ischaemia, particularly in the thalamus, basal ganglia and internal capsule [96]. Tuberculomas arise anywhere in the brain, but around half of parenchymal tuberculomas are situated peripherally and have a close relation to the dura, sometimes appearing to have a dural attachment [97].

Diagnostic uncertainty usually occurs in cases of patients with isolated dural lesions and a relatively mild clinical picture [93, 98–100]. Reduced or even absent meningeal enhancement has been reported in cases of acquired immune deficiency syndrome (AIDS) where the immune response to infection is impaired, causing further difficulty [97]. Elderly patients may lack the typical basal exudates, infarcts and tuberculomas seen in younger patients, also due to reduced immune activity [101].

On MRS, lesions demonstrate decreased NAA:Cr and NAA:choline, with lipid-lactate peaks also being elevated in 86% due to areas of necrosis [102]. Diffusion characteristics are variable and can be similar to metastases, with tuberculomas being less restricting than true tuberculosis abscesses [103]. Perfusion techniques show tuberculous lesions have rCBV values that are similar to or lower than most metastases and therefore also significantly lower than meningiomas [103].

### Granulomatosis with polyangiitis

Granulomatosis with polyangiitis(GPA), previously known as Wegener’s granulomatosis, is a rare systemic disorder which usually begins with necrotising granulomatous inflammation within the respiratory tract and small to medium vessels [104]. More than 90% of patients have anti-neutrophil cytoplasmic antibodies (ANCA), with the diffuse cytoplasmic (cANCA) staining at indirect immunofluorescence associated with severe disease and peripheral staining (pANCA) with more limited diseases [105]. CNS involvement is seen in 20–50% of cases, with meningeal disease only reported in around 1% [106]. Interestingly, this meningeal involvement is usually found in those patients with more limited clinical disease as well as upper respiratory tract involvement [106].

CNS involvement is thought to occur by several mechanisms: intracranial vasculitis; involvement of the skull base or nearby structures via direct spread from the paranasal sinuses; or independent granulomatous inflammation of the meninges or brain, without direct spread from the respiratory tract [104]. Clinical presentation is typically with seizures, meningism, cranial nerve palsy or ischaemic phenomena due to intracranial vasculitis. Treatment is aimed at reducing inflammation with immunosuppressive agents such as glucocorticoids and cyclophosphamide.

#### Imaging features

**Fig. 12:**
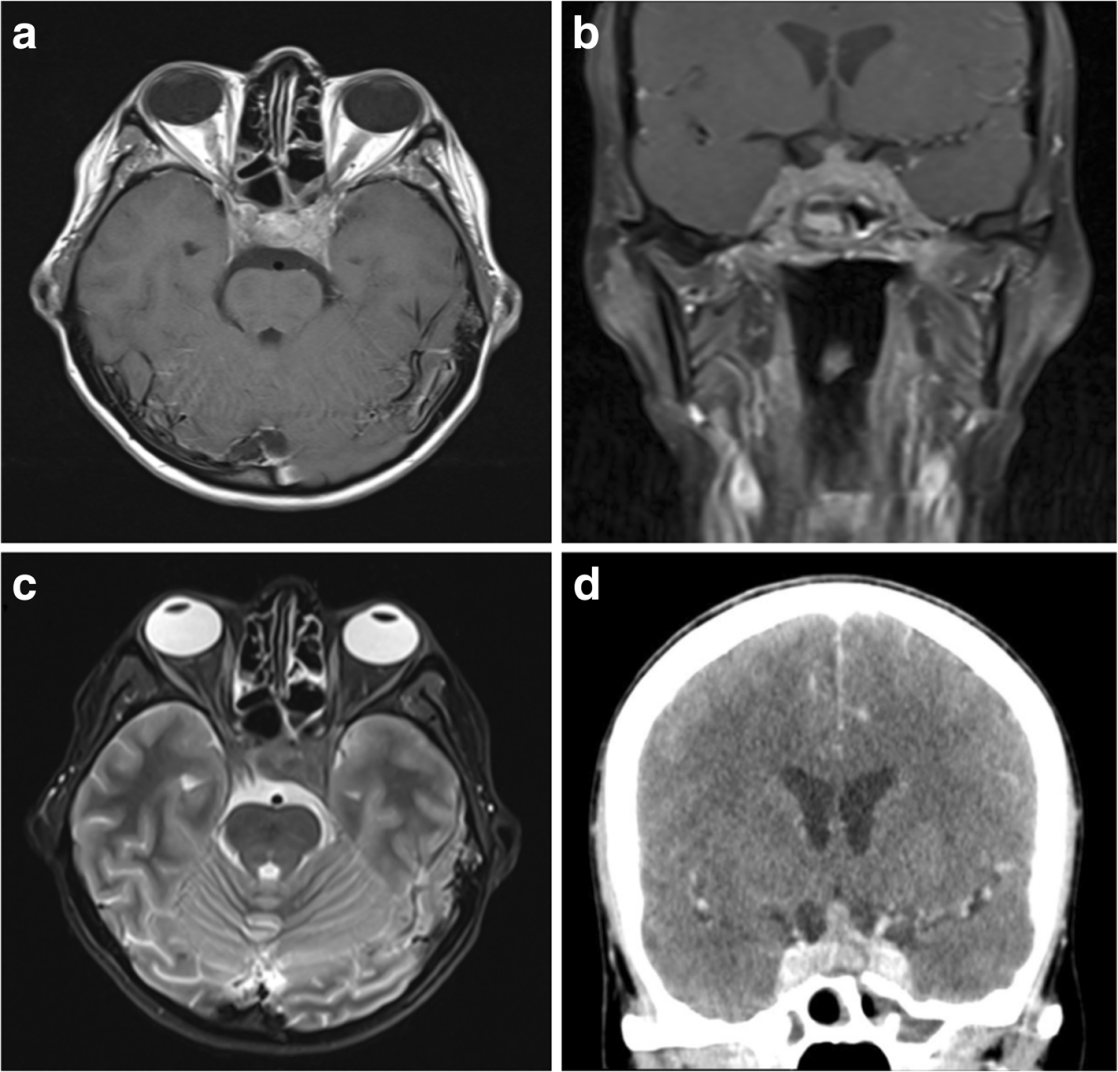
Granulomatosis with polyangiitis of the skull base. **a**, **b** Post-contrast T1-weighted axial and coronal MR images showing enhancing inflammatory soft tissue involving the skull base and cavernous sinuses. There is also inflammatory change within the adjacent sphenoid sinus. **c** T2-weighted axial image of the same lesion at the level of the cavernous sinuses. **d** Post-contrast axial CT image again showing avid enhancement within durally based abnormal soft tissue

Meningeal disease manifests as either diffuse dural thickening (73%) or focal mass-like lesions (27%), with the leptomeninges also involved in around a third of patients [106]. Focal dural lesions are caused by direct involvement from paranasal sinus or orbital disease (Fig. 12), while diffuse meningeal thickening tends to be a separate granulomatous process [107].

On CT, areas of dural thickening are isodense and in longstanding disease may develop intralesional calcification [108]. CT is also useful for assessing the adjacent paranasal sinuses for invasive granulomatous disease which may support the underlying diagnosis, particularly if bilateral [109]. Paranasal lesions may invade directly into other adjacent structures such as the Eustachian tubes and bone [109]. Dural lesions are isointense on T1WI and T2WI and demonstrate moderate post-contrast enhancement [107]. Diffusion-weighted imaging shows restricted diffusion within areas of dural thickening and is also useful for assessing for secondary cerebral ischaemia relating to vasculitis [109, 110].

Other accompanying manifestations in the head and neck include orbital inflammatory pseudotumours, as well as lacrimal and salivary gland enlargement [109].

### Epstein–Barr virus-associated smooth muscle tumours

The Epstein–Barr virus (EBV) which belongs to the human herpesvirus family has been associated with the development of a number of malignancies including Burkitt’s lymphoma, non-Hodgkin lymphoma and nasopharyngeal cancers [111]. Infection appears to be a requisite step in the development of a group of smooth muscle tumours arising exclusively in immunocompromised patients such as those with acquired immune deficiency syndrome (AIDS), and those who have undergone solid organ transplantation. EBV-associated smooth muscle tumours (EBV-SMT) may arise in almost any body tissue, having been reported in the colon, liver, adrenal glands, and soft tissues; however, they occur most frequently in the CNS in an extra-axial location. A large case review in AIDS patients showed patients presenting with EBV-SMTs to have modest levels of immunosuppression, with mean CD4 counts of 60 cells/μL, although in some cases above 200 cells/μL [111]. The time from HIV diagnosis to EBV-SMT presentation ranged from under 1 month to 18 years, with two thirds arising in the first 4 years. Benign leiomyomas and malignant leiomyosarcomas have been reported, and while multifocal lesions are the norm, these appear to arise more often as separate neoplasms rather than metastasising. Treatment is primarily with resection although cases in the literature have been managed with a combination of chemotherapy, radiotherapy and highly active antiretroviral therapy (HAART) [111]. EBV-SMT should be considered in any immunodeficient patient with dural lesions especially if they are multifocal.

#### Imaging features

EBV-SMTs arise most commonly as multifocal dural masses which occur within the cranium and the spine [112]. On CT, lesions are hyperdense to cortex, enhance avidly and are commonly found with erosion of the adjacent skull. Intralesional calcification may also be present.

On MRI, lesions are isointense to hypointense on T2WI, sometimes containing foci of high signal [112]. On T1WI, they are isointense to hypointense, enhance strongly and may have a dural tail. They do not typically demonstrate restricted diffusion [112].

Tumours have been shown to demonstrate avid tracer uptake on FDG PET imaging, which is useful for detecting systemic lesions which are often multifocal [113].

### IgG4-related disease

IgG4-related disease (IgG4-RD), also known as IgG4-related sclerosing disease, is a recently described inflammatory disorder which was only recognised as a systemic condition when patients with autoimmune pancreatitis (AIP) were found to have raised serum levels of IgG4 as well as additional extrapancreatic manifestations [114]. Resected pancreatic tissue in these patients also demonstrated inflammatory infiltrates containing IgG4-positive plasma cells and lymphocytes with fibrosis and obliterative phlebitis. Since then, similar IgG4-positive fibroinflammatory changes have been found in many other conditions including Sjögren syndrome, sclerosing cholangitis, primary biliary cirrhosis and multifocal fibrosclerosis. IgG4-RD typically affects men in their fifth to sixth decade, and the most commonly presenting lesion is tumefactive or mass-like with simple infiltration occurring less commonly [115].

CNS involvement is rare. IgG4-RD has been described in cases of hypophysitis, and there are also descriptions of inflammatory pseudotumour and IGG4-RD-associated hypertrophic pachymeningitis [116]. There is usually synchronous or metachronous systemic involvement and meningeal lesions have been described intracranially as well as the spine. Tissue sampling is required for definitive diagnosis. While there are currently no internationally recognised histopathological criteria in meningeal disease, similar criteria to AIP (> 10 IgG4-positive cells per high power field) appear appropriate and have been recommended by some authors [117]. Despite this, aetiology is often initially inferred when active systemic disease has been elicited and other causes of meningeal disease have been excluded [117]. The disease usually responds well to steroids, and this forms the mainstay of treatment [118].

#### Imaging features

Several patterns of CNS involvement have been described: meningeal disease follows a hypertrophic pachymeningitis pattern and manifests as either *diffuse linear* dural thickening over the cerebral convexities and skull base, or more often with *focal nodular* dural lesions [115]. The cranial nerves are often involved, with concentric thickening mimicking cranial nerve sheath meningiomas. Additional findings which may be encountered in the head and neck include hypophysitis, intraorbital pseudotumours, salivary and lacrimal gland enlargement, as well as pituitary and thyroid lesions [119].

**Fig. 13:**
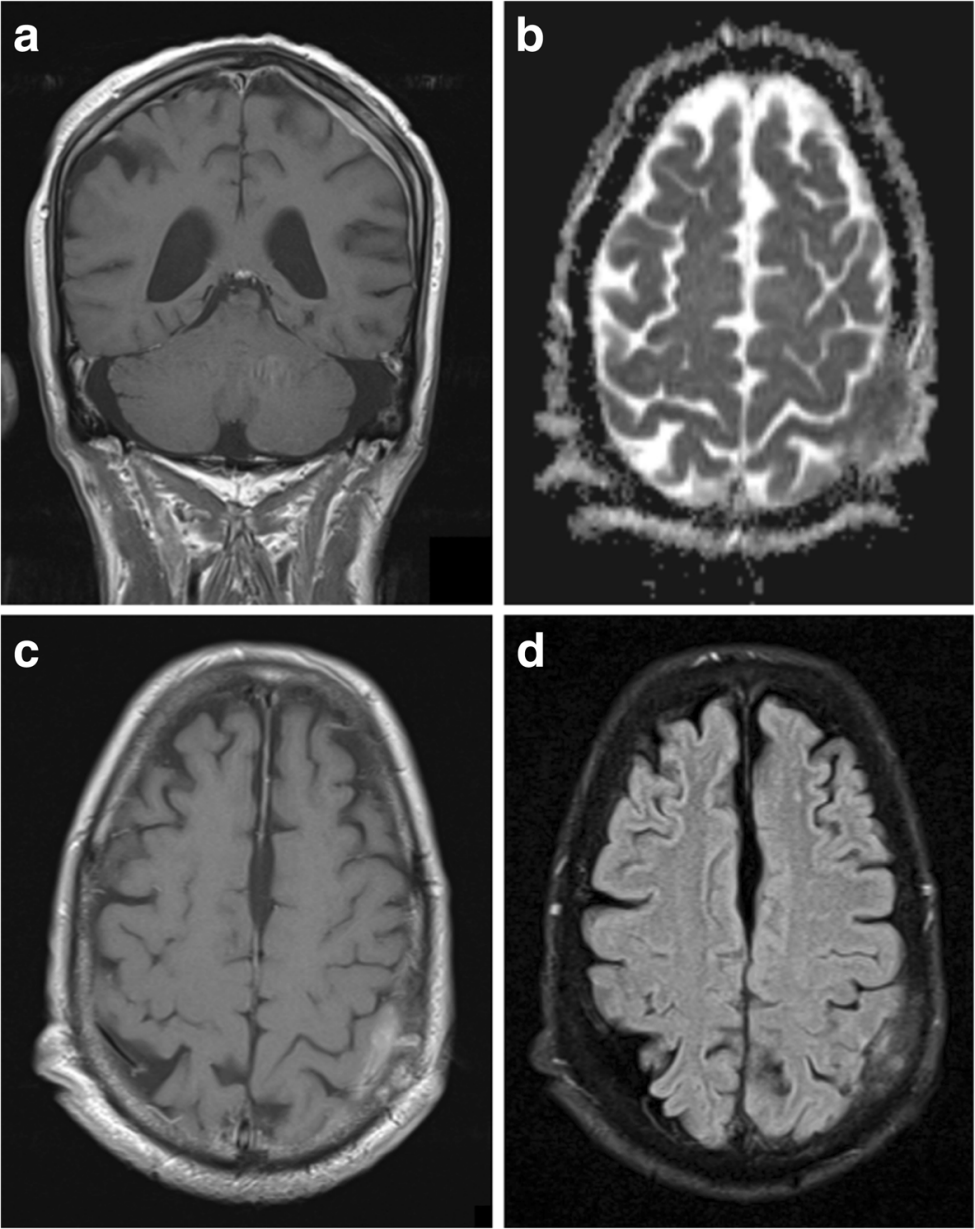
IgG4-related hypertrophic pachymeningitis with skull invasion. **a** Post-contrast coronal T1-weighted MR image showing linear dural thickening and enhancement overlying the left cerebral convexity. Note that the overlying calvarial bone marrow signal is abnormal. **b** Apparent diffusion coefficient (ADC) map showing restricted diffusion in the lesion overlying the left parietal region. **c** Post-contrast axial T1-weighted image of the same lesion with enhancing soft tissue seen invading the skull. **d** Axial fluid attenuated inversion recovery (FLAIR) image showing the lesion is predominantly hypointense due to fibrosis with foci of hyperintensity

Lesions are hypointense on T1WI with homogeneous avid enhancement and may be accompanied by a dural tail [119]. In contrast to meningiomas, they are markedly hypointense on T2WI and gradient echo sequences such as SWI due to intralesional fibrosis. Scattered foci of hyperintensity can be seen on T2WI and FLAIR imaging due to areas of increased intralesional inflammation (Fig. 13d) [120]. On CT imaging, lesions are of hyperdense soft tissue density, and no intralesional calcification has yet been described. Lesions can cause some remodelling of adjacent bone and very rarely bone infiltration and destruction may occur [121]. Dynamic contrast-enhanced CT has been reported to show sluggish enhancement [122].

While there is no detailed description in the literature of the advanced MRI characteristics of dural IgG4-RD, most systemic lesions as well as intraorbital pseudotumours demonstrate restricted diffusion due to their fibrotic constituents [123]. This would fit with the findings in our case of IgG4-related hypertrophic pachymeningitis (Fig. 13).

Lesions demonstrate increased metabolic activity on FDG PET, and this is useful for assessment of systemic disease as well as treatment response [124]. However, due to the high degree of FDG tracer uptake in normal brain tissue, assessment of intracranial disease with alternative tracers such as methionine PET is again preferred [125].

## Conclusion

Many pathologies affecting the dura can mimic meningiomas. These include primary neoplastic processes, as well as inflammatory, infectious and metastatic disease. While differentiation is difficult on imaging alone, there are many characteristic features which may help point to the diagnosis (Table 2). In contrast to meningiomas, many of these lack intratumoural calcification (except for low grade SFT) and are associated with bony erosion rather than hyperostosis or sclerosis. The dural tail sign which is considered a characteristic feature of meningiomas should be evaluated with caution as it is a feature of many other dural processes. Meningiomas are hypervascular and have higher perfusion values compared with most other extra-axial lesions with the exception of hypervascular metastases such as those of renal carcinoma. MRS, while not currently used routinely in clinical practice, is useful in differentiating meningiomas in some cases with high alanine and low NAA in meningiomas, high lipid/lactate in metastases and high myoinositol in solitary fibrous tumours.

**Table 2:** Summary of the imaging findings in meningiomas and their mimics

	Summary of typical imaging findings	Useful distinguishing features
Meningioma	CT: hyperdense, wide base, often with intratumoural calcification. T1WI: isointense. T2WI: isointense. Other features: avid enhancement with enhancing dural tail, adjacent hyperostosis, ECA blood supply.	Intratumoural calcification. Hyperostosis of adjacent skull. May have sunburst enhancement due to internal vessels. High choline:Cr ratio, low NAA peak on MRS. Alanine peak, if present, is characteristic. High rCBV compared with most mimics
Metastasis	CT: hyperdense, diffuse thickening or mass like. T1WI: hypointense but variable. T2WI/FLAIR: hypointense but variable. Other features: strongly enhancing. Local invasion with destruction of the bone. Hypervascular metastases (renal and melanoma) can have similar rCBV to meningiomas.	Can be hypointense on T2WI. Multivessel blood supply from ICA and ECA. Invasion of adjacent skull and soft tissue structures. Low NAA:Cr and high lipid:Cr ratio on MRS.
Low-grade SFT	CT: iso- to hyperdense. Well circumscribed. Can contain calcification. T1WI: isointense T2WI: mixed signal due to hypointense regions of collagen. Other features: solitary. Even hypointense areas on T2WI enhance. Separate regions of different T2 signal can cause Yin-Yang sign. May have high rCBV similar to meningiomas.	May have ‘fluffy’ contrast enhancement rather than sunburst. Enhancement of regions of hypointensity on T2WI (collagen). High myo-inositol with lipid and lactate peaks on MRS.
High-grade SFT	CT: hyperdense. May ‘mushroom’ away from the dura with due to a narrow dural base T1WI: heterogeneous, with areas of haemorrhage. T2WI/FLAIR: heterogeneous with areas of cystic change and necrosis. Other features: heterogeneous strong enhancement. Higher grades are more likely to cause bone destruction and cross the midline. A dural tail is common but less so in the highest-grade lesions. Multiple flow voids. Solitary. May have high rCBV similar to meningiomas.	No calcification. Destruction of the bone rather than hyperostosis. Often a narrow dural base. Highly vascular with supply from ICA and ECA. High myo-inositol on MRS.
Lymphoma	CT: hyperdense. T1WI: hypointense. T2WI/FLAIR: iso- to hypointense. Other features: may be lobulated with indistinct ‘fluffy borders’. Strong enhancement and prominent perilesional oedema. Secondary leptomeningeal disease can cause meningeal thickening and enhancement.	No calcification and no hyperostosis. Lobulated Fuzzy tumour-brain interface
Melanotic tumours	CT: iso- to hyperdense. May be diffuse or mass like. T1WI: contain areas of hyperintensity due to haemorrhage and high melanin content. T2WI/FLAIR: iso- to hypointense. Other features: strongly enhancing. Lack of primary systemic melanotic lesion is suspicious for primary CNS disease.	No calcification and no hyperostosis. Usually contain areas of T1 hyperintensity due to melanin and haemorrhage
Gliosarcoma	CT: heterogeneous mass with hyperdense soft tissue components T1WI: isointense but may contain areas of haemorrhage T2WI: heterogeneous with areas of necrosis Other features: heterogeneous or peripheral strong enhancement, small amount of perilesional oedema.	High lipid and high lipid-choline ratio on MRS Some lesions have peripheral enhancement. No calcification and no hyperostosis.
Rosai-Dorfman disease	CT: hyperdense. Well circumscribed with no calcification. T1WI: isointense T2WI/FLAIR: iso- to hypointense. Other features: erosion of adjacent bone with slight vasogenic oedema. Arise from meninges or endocranium.	More likely to have bone erosion with no calcification or hyperostosis. Lesions are hypovascular.
Erdheim-Chester disease	CT: hyperdense. Nodular or diffuse meningeal thickening. T1WI: isointense. T2WI/FLAIR: iso- to hypointense. Other features: strong, homogenous enhancement that can persist for up to 8 days. Most patients have diffuse calvarial thickening or paranasal sinus wall osteosclerosis.	Patients with intracranial disease will almost always have facial bone or calvarial sclerosis or thickening. Persistent dural enhancement.
Tuberculosis	CT: iso- to hyperdense. Focal nodular or diffuse dural thickening. T1WI: isointense. T2WI/FLAIR: isointense. Other features: strong enhancement without calcification. Parenchymal tuberculomas can also attach to dura and can have solid or ring-enhancement.	Meningitis preferentially affects the basal meninges. No calcification in acute phase. Erosion of the bone instead of hyperostosis. Peripheral parenchymal lesions may have ring-enhancement rather than solid. Decreased NAA:Cr and NAA:choline ratios.
Granulomatosis with polyangiitis	CT: isodense diffuse or focal dural thickening. T1WI: isointense. T2WI/FLAIR: isointense. Other features: moderate uniform enhancement. Focal nodular disease typically affects the skull base and tends to be contiguous with paranasal sinus involvement. Ischaemic changes in the brain due to vasculitis. Longstanding disease may calcify.	Characteristically located at the skull base. Contiguous invasive inflammatory soft tissue in adjacent paranasal sinuses. Secondary features of cerebral vasculitis.
Epstein–Barr virus-associated smooth muscle tumour	CT: hyperdense dural masses. T1WI: iso- to hypointense. T2WI/FLAIR: iso- to hypointense. Other features: avid enhancement. Multifocal.	Patients are immunosuppressed, most commonly due to AIDS or after solid organ transplantation. Typically multifocal with additional CNS and systemic lesions arising in many tissues.
IgG4-related disease	CT: hyperdense. Focal nodular or diffuse linear dural thickening. T1WI: hypointense. T2WI/FLAIR: hypointense. Sometimes additional foci of hyperintensity Other features: avid homogeneous enhancement. Typically causes remodelling of adjacent bone but infiltration also very rarely occurs. Avid FDG and methionine PET tracer uptake.	Often found in the presence of other IgG4-related diseases, and patients characteristically have raised serum IgG4. Lesions are low signal on T1WI and T2WI due to fibrosis.

*AIDS* acquired immune deficiency syndrome, *CT* computed tomography, *Cr* creatine, *CNS* central nervous system, *ECA* external carotid artery, *FLAIR* fluid attenuated inversion recovery, *ICA* internal carotid artery, *IgG4* immunoglobulin G4, *MRS* magnetic resonance spectroscopy, *NAA* N-acetylaspartate, *rCBV* relative cerebral blood volume, *T1WI* T1-weighted imaging, *T2WI* T2-weighted imaging, *SFT* solitary fibrous tumour
